# Psychometric properties of an Arabic translation of the 10-item Connor-Davidson resilience scale (CD-RISC-10), the 8- and 10-item post-traumatic growth inventory-short form (PTGI-SF) scales

**DOI:** 10.1371/journal.pone.0293079

**Published:** 2024-01-02

**Authors:** Feten Fekih-Romdhane, Mirna Fawaz, Rabih Hallit, Toni Sawma, Sahar Obeid, Souheil Hallit

**Affiliations:** 1 The Tunisian Center of Early Intervention in Psychosis, Department of Psychiatry “Ibn Omrane”, Razi Hospital, Manouba, Tunisia; 2 Tunis El Manar University, Faculty of Medicine of Tunis, Tunis, Tunisia; 3 Faculty of Health Sciences Beirut Arab University, Tareek Al Jadida, Afeef Al Tiba, Beirut, Lebanon; 4 School of Medicine and Medical Sciences, Holy Spirit University of Kaslik, Jounieh, Lebanon; 5 Department of Infectious Disease, Bellevue Medical Center, Mansourieh, Lebanon; 6 Department of Infectious Disease, Notre Dame des Secours University Hospital, Byblos, Lebanon; 7 School of Arts and Sciences, Social and Education Sciences Department, Lebanese American University, Jbeil, Lebanon; 8 Applied Science Research Center, Applied Science Private University, Amman, Jordan; 9 Research Department, Psychiatric Hospital of the Cross, Jal Eddib, Lebanon; University of São Paulo, BRAZIL

## Abstract

**Background:**

Given their clinical significance and impact on stress response and their potential malleability, resilience and posttraumatic growth (PTG) should receive greater attention as relevant constructs in clinical and research practice in the Arab context. We aimed through the present study to test the psychometric properties of Arabic translations of the 10-item Connor-Davidson Resilience scale (CD-RISC-10), the 10-item and the 8-item Post-Traumatic Growth Inventory-Short Form (PTGI-SF) in a sample of Lebanese adults from the general population.

**Methods:**

Three hundred eighty-seven Arabic-speaking participants (mean age = 26.17; 58.4% females) responded to a self-report web-based questionnaire. The forward and backward translation method was applied with the approval of the original developers of the scales.

**Results:**

Confirmatory factor analysis indicated that fit of the one-factor model was acceptable, and all indices suggested that configural, metric, and scalar invariance was supported across gender for all the three scales. The CD-RISC-10, the 10-item and the 8-item PTGI-SF yielded a good internal consistency, with a McDonald’s ω of .89, .95, and .93, respectively. Higher resilience and higher PTG were significantly and positively associated with greater cognitive reappraisal and lower emotion suppression, supporting convergent validity.

**Conclusion:**

We preliminarily suggest that these Arabic instruments are appropriate for use in Lebanese community adults to assess different positive responses after life crises, identify people with lack or low levels of resilience and growth who might need intervention, and monitor their response to therapy. Further cross-cultural validations should seek to extend their use in broader Arabic-speaking populations and settings.

## Background

Exposure to trauma not only contributes to harmful mental health impact, but may also be associated with continued normal functioning or even induce positive changes [1]. The latter outcomes may be attributable to positive dynamic psychological processes, such as post-traumatic growth (PTG) and resilience [2, 3]. Indeed, individuals substantially differ in the ways how they face challenges and adversity. While struggling to heal after traumatic and stressful events, an individual may actively develop positive emotional experiences, personal growth, and transform in a way that positively affects their life, known as PTG [4]. However, when adversity or trauma strike, they do not always make individuals struggle or shake their beliefs, but can rather enable them to simply bounce back, known as resiliency [5]. The presence of resilience and PTG is associated with positive indicators of mental health (e.g., self-esteem [6, 7], perceived social support [6, 8], positive affect [9, 10], sleep quality [11–13]), and are manifested in greater well-being [14], whereas when lacking, they are related to a range of mental health and behavioral problems [15–17]. Interestingly, resilience and PTG have proven to be modifiable through interventions [18]. A critical step in assessing the efficiency of such interventions is the use of measurement instruments. Therefore, to prevent negative outcomes and promote mental health and wellbeing after exposure to traumatic or stressful life events, valid and reliable assessment measures are needed. Such measures will enable sound assessment, monitoring and identification of target populations under adversity who are in need of enhanced interventions. Valid and reliable measures can foster future research on resilience in Arab countries, and help design and implement culturally-tailored and evidence-informed interventions.

To this end, Connor and Davidson developed a 25-item five-factor measure, the Connor-Davidson Resilience Scale (CD-RISC), which assesses resilience through self-report [5]. Despite being one of the best three resilience scales in terms of psychometric quality [19] and the most widely used worldwide, this original English version of the CD-RISC has demonstrated unstable psychometric properties across societies and cultures (i.e., inconsistent number of items [20–22] and/or factors [23–26]). This motivated many researchers to develop shorter and more psychometrically sound versions (10 and 2 items) [27], from which the most stable and most reliable at capturing the resilience construct is the 10-item version (CD-RISC-10) [28]. The CD-RISC-10 has been translated into different languages, including French [29], Spanish [30, 31], Finnish [32], Afrikaans [33], Persian [34], and Chinese [35]; it has also been tested in different specific populations, i.e., students [33, 35, 36], community young adults [30, 31, 37], older adults [32, 38, 39], and adults on the autism spectrum [40]. The CD-RISC-10 yielded a unidimensional factor structure and good psychometric properties in both the original [28] and other linguistic [29–32, 34–36] validation studies. However, an Arabic shortened version of the CD-RISC has not been developed so far to the best of our knowledge.

On the other hand, the gold standard for the assessment of PTG is the Post-Traumatic Growth Inventory (PTGI), a six-point 21-item scale that has been developed by Tedeschi and Calhoun [41]. The PTGI measures positive changes following trauma. Since this original version published in 1996, there has been a few attempts to develop shortened versions of the PTGI including a 10-item five-factor structure version by Cann et al. (Posttraumatic Growth Inventory-Short Form; PTGI-SF) [42], an 8-item four-factor structure version by Garrido-Hernansaiz et al. [43], and a 5-item one-factor structure version by Gómez-Acosta et al. [44]. The PTGI has been largely used to thoroughly investigate PTG in various populations and cultures [45]. In the present work, we are interested in the 10-item and 8-item PTGI-SF, which have been translated in several languages including Spanish [43, 46], Portuguese [47], French [48], Italian [49], Urdu [50], Malay [51], and Persian [52]. It is of note that the dimensionality of PTG construct has been questioned, with studies describing the PTGI as being either unidimensional (e.g., [53]), or as being formed by three- (e.g., [54]), four- [55, 56], and five- [57] factors. A plausible explanation of these differences may be cultural factors, as the multidimensional structure was observed in highly individualistic cultures (e. g., Australia [57]), while a single dimension tended to appear in more collectivist countries [58]. The 10-item PTGI-SF has been validated in the Arabic language in a sample of Palestinian professional helpers [59], however, Palestinians represent a specific and particularly vulnerable population due to their prolonged exposure to political violence, war experiences, and mental healthcare shortages [60], and might not be representative of the broader Arabic-speaking population. This suggests that its validation in another Arab context is needed to confirm its cross-cultural validity.

### The Arab context

The ecological interpretation of resilience by Ungar [61] informs a definition of the construct based on the four following principles: (1) Decentrality, which implies that, when facing adversity, changes reside not only in individual resources, but also in the processes by which the environment provide resources for the individual; (2) Complexity, which refers to the fact that changes that occur under adversity and their causal processes are too complex, with aspects of the environment being likely to exert more influence on outcomes than individual traits, (3) Atypicality, which suggests that resilience may manifest in unusual ways (such as atypical use of resources manifesting in nondemocratic decision making in a family) that are culturally and contextually relevant for adaptive and positive changes, and (4) Cultural Relativity, which reflects the extent to which processes of changes under adversity are both temporally (historically) and culturally embedded. Overall, this social ecological model of resilience suggests that resilience is “less an individual trait” and more a quality of the individual’s physical and social ecology [61]. Therefore, this ecological understanding of growth and resilience shifts the focus from the individual to the changing environments, and emphasizes the major importance of a better understanding of the processes that are likely to foster resilience within a given culture and context. The Palestinian socio-political concept of ‘Sumud’ is a perfect example illustrating the new conceptual framework of the social ecology of resilience [62], and refers to ways of surviving in the specific Arab Palestinian context of chronic adversity, ongoing occupation, limited infrastructure and lack of resources. Lebanese youth facing a series of crises and disastrous living conditions [63] and war-exposed Syrians [64] represent two other examples of resilience largely involving physical/social ecologies and particular cultures/environments that are far different from those observed in the Western world. However, there is scant data on resilience within conflict zones and in underdeveloped countries, and a very limited amount of research on resilience within an Arabic or Muslim cultural context [62]. During the last decades, Arab countries have undergone a series of conflicts, wars and terrorist attacks [65], and faced, like all other countries, increasing disaster and climate-related threats [66, 67]. Nevertheless, most of the research literature emerging from the Arab world have focused on the negative psychological effects of experiencing these crises (e.g., [68–73]), while only very few studies addressed resilience and PTG [74–77]. This highlights the importance of investigating and deepening the understanding of positive human responses to trauma and stress in these specific contexts. Indeed, given their clinical significance and impact on stress response and their potential malleability, resilience and PTG should receive greater attention as relevant constructs in clinical and research practice in Arab contexts. As such, there is a strong need to provide clinicians and researchers with standardized methods and valid, reliable scales in the Arabic language to evaluate these constructs.

### The present study

Validating the short forms of the CD-RISC and the PTGI for Arabic-speaking people is valuable for more than one reason. First, resilience and PTGI are culturally-dependent constructs [78, 79], and may be subject to change according to personal contexts and circumstances [5]. As such, developing brief, easy-to-use measures in the Arabic language can help promote research on these constructs in the under-researched Arab-speaking populations, and therefore enhance our knowledge of how resilience and PTGI models differ across cultures, contexts and groups [78]. Second, the target population of these scales (i.e., CD-RISC and PTGI) are generally survivors of major life crises (e.g., cancer, natural disasters, wars, terrorism), who are likely to lack time and energy when responding to the questionnaire [42]. Third, administering a reduced number of items implies lower costs of data gathering, which is of high interest economically in the low-middle income Arab countries. Fourth, the available CD-RISC and PTGI adaptations are not completely homologous to each other across countries, cultures and settings. Given that there is no Arabic version of the CD-RISC-10 and the 8-item PTGI-SF, and the fact that PTGI-SF has only been validated in a narrow context and a particular population (i.e., Palestinian health providers), the need for an Arabic translation of these instruments in an Arabic-speaking population with extended sociodemographic and cultural characteristics is evident.

We aimed through the present study to test the reliability and validity of Arabic translations of the CD-RISC-10, the 10-item and the 8-item PTGI-SF in Lebanese adults from the general population, with the approval of the original developers. We hypothesized that the Confirmatory Factor Analysis (CFA) would show that the theoretically assumed one-factor model of the CD-RISC-10 will fit the data well. Since Arab countries are collectivist in nature [80], we also hypothesized that the 10-item and the 8-item PTGI-SF will yield a unidimensional factor structure. In addition, we expected that the three scales will show good internal consistency, measurement invariance across gender, and that their total scores will be positively correlated with emotion regulation, which corresponds to the way how individuals shape, experience and express their emotions [81].

## Methods

### Procedures

Ethics approval for this study was obtained from the Psychiatric Hospital of the Cross ethics committee (approval code: HPC-023-2022). Written informed consent was obtained from all subjects; the online submission of the soft copy was considered equivalent to receiving a written informed consent. All data were collected via a Google Form link, between May and July 2022. The project was advertised on social media and included an estimated duration. Inclusion criteria for participation included being a resident and citizen of Lebanon of adult age. Excluded were those who refused to fill out the questionnaire. The “data duplicate” option in excel was used to ensure that the same answer was not recorded more than once. After providing digital informed consent, participants were asked to complete the instruments described below. Consent was assumed if a person completed the survey in its entirety. The survey was anonymous and participants completed the survey voluntarily and without remuneration.

### Translation procedure

The forward and backward translation method was applied to the resilience and post-traumatic stress scales. The English version was translated to Arabic by a Lebanese translator who was completely unrelated to the study. Afterwards, a Lebanese psychologist with a full working proficiency in English, translated the Arabic version back to English. The initial and translated English versions were compared to detect and later eliminate any inconsistencies by a committee composed of the research team and the two translators.

### Measures

#### Connor-Davidson Resilience Scale (CD-RISC)

The CD-RISC comprises 10 items [28, 82], each of which are scored on a 5-point scale ranging from 0 (not true at all) to 4 (true nearly all of the time). Examples of items include, “I am able to adapt when changes occur” and “I am not easily discouraged by failure.” Higher scores on the CD-RISC-10 indicate higher levels of resilience.

#### Post traumatic growth Inventory-Short Form (PTGI-SF)

This scale measures favourable outcomes after a traumatic event, including 5 dimensions: relating to others, new possibilities, personal strength, spiritual change and appreciation of life. Two versions exist for this scale, the 10-item one [83] and the 8-item one [43].

#### Emotion regulation questionnaire

Validated in Lebanon [84], it is a ten-item tool used to assess emotion regulation, which yields two scores: cognitive reappraisal and emotion suppression. Higher scores indicate higher aspects of emotion regulation respectively (McDonald’s ω was .84 for emotion suppression and .91 for cognitive reappraisal in this study).

#### Demographics

Participants were asked to provide their demographic details consisting of age, sex, highest educational attainment, region of living, marital status and the Household Crowding Index (HCI); the latter reflecting the socioeconomic status (SES) of the family [85], is the ratio of the number of persons living in the house over the number of rooms in it (excluding the kitchen and the bathrooms). Values <1 reflect a high SES, between 1–2 a moderate SES and >2 a low SES.

### Analytic strategy

#### Confirmatory factor analysis

There were no missing responses in the dataset. We used data from the total sample to conduct a CFA using the SPSS AMOS v.26 software. The minimum sample size to conduct a confirmatory factor analysis ranges from 3 to 20 times the number of the scale’s variables [86]. Therefore, we assumed a minimum sample of 200 participants needed to have enough statistical power based on a ratio of 20 participants per one item of the scale, which was exceeded in our sample. Our intention was to test the original model of the resilience and PTG scores (i.e., unidimensional models). Parameter estimates were obtained using the maximum likelihood method and fit indices. To check if the model was adequate, several fit indices were calculated: the normed model chi-square (χ^2^/df), the Steiger-Lind root mean square error of approximation (RMSEA), the Tucker-Lewis Index (TLI) and the comparative fit index (CFI). Values ≤ 5 for χ^2^/df, and ≤ .08 for RMSEA, and .95 for CFI and TLI indicate good fit of the model to the data [87]. Additionally, evidence of convergent validity was assessed in this subsample using the Fornell-Larcker criterion, with average variance extracted (AVE) values of ≥ .50 considered adequate [88].

#### Gender invariance

To examine gender invariance of CD-RISC-10 and PTGI-SF scores, we conducted multi-group CFA [89] using the total sample. Measurement invariance was assessed at the configural, metric, and scalar levels [90]. Configural invariance implies that the latent scales variable(s) and the pattern of loadings of the latent variable(s) on indicators are similar across gender (i.e., the unconstrained latent model should fit the data well in both groups). Metric invariance implies that the magnitude of the loadings is similar across gender; this is tested by comparing two nested models consisting of a baseline model and an invariance model. Lastly, scalar invariance implies that both the item loadings and item intercepts are similar across gender and is examined using the same nested-model comparison strategy as with metric invariance [89]. We accepted ΔCFI ≤ .010 and ΔRMSEA ≤ .015 or ΔSRMR ≤ .010 as evidence of invariance [89, 91].

#### Further analyses

Composite reliability in both subsamples was assessed using McDonald’s ω, with values greater than .70 reflecting adequate composite reliability [92]. McDonald’s ω was selected as a measure of composite reliability because of known problems with the use of Cronbach’s α (e.g., [93]). To assess convergent and criterion-related validity, we examined bivariate correlations between resilience and PTG scores and emotion regulation. Based on Cohen (1992) [94], values ≤ .10 were considered weak, ~ .30 were considered moderate, and ~ .50 were considered strong correlations.

## Results

Three hundred eighty-seven participants participated in this study, with a mean age of 26.17 ± 11.47 years and 58.4% females. The mean and standard deviations of the scores used were as follows: resilience (23.88 ± 7.29), PTG-10 (28.04 ± 11.78), PTG-8 (22.48 ± 9.39), cognitive reappraisal (23.83 ± 16.56), and expressive suppression (8.36 ± 5.51). Other descriptive statistics of the sample can be found in Table 1.

**Table 1:** Sociodemographic and other characteristics of the sample (N = 387).

Variable	N (%)
Sex	
Male	161 (41.6%)
Female	226 (58.4%)
Marital status	
Single	311 (80.4%)
Married	76 (19.6%)
Education level	
Secondary or less	66 (17.1%)
University	321 (82.9%)
Region of living	
Urban	294 (76.0%)
Rural	93 (24.0%)
	**Mean ± SD**
Age (years)	26.17 ± 11.47
Household crowding index (persons/room)	1.47 ± 1.00

### Confirmatory factor analysis of the resilience scale

CFA indicated that fit of the 1-factor model of the CD-RISC-10 was acceptable: χ^2^/df = 142.69/35 = 4.07, RMSEA = .089 (90% CI = .074, .105), SRMR = .047, CFI = .931, TLI = .912. When adding a correlation between residuals 1 and 2, the fit indices improved as follows: χ2/df = 103.49/34 = 3.04, RMSEA = .073 (90% CI .057, .089), SRMR = .043, CFI = .956, TLI = .941. The standardised estimates of factor loadings were all adequate (see Table 2). The convergent validity for this model was borderline, as AVE = .44.

**Table 2:** Items of the CD-RISC-10 in English and factor loadings derived from the confirmatory factor analysis (CFA) in the total sample.

Item	Total
1. I am able to adapt to change.	.56
2. I can deal with whatever comes.	.75
3. I try to see the humorous side of problems.	.45
4. Coping with stress can strengthen me.	.67
5. I tend to bounce back after illness or hardship.	.72
6. I can achieve goals despite obstacles.	.72
7. I can stay focused under pressure.	.62
8. I am not easily discouraged by failure.	.59
9. I think of myself as a strong person.	.77
10. I can handle unpleasant feelings.	.71

### Gender invariance

As reported in Table 3, all indices suggested that configural, metric, and scalar invariance was supported across gender. Given these results, we computed an independent-samples t-test to examine gender differences in CD-RISC-10 scores. The results showed that there was no statistically significant difference between men (M = 24.54, SD = 7.69) and women (M = 23.40, SD = 6.98), t(385) = 1.52, p = .131, d = 2.71.

**Table 3:** Measurement invariance across gender in the total sample.

Model	χ^2^	df	CFI	RMSEA	SRMR	Model Comparison	Δχ^2^	ΔCFI	ΔRMSEA	ΔSRMR	Δdf	p
Configural	160.38	68	.943	.059	.044							
Metric	174.06	77	.940	.057	.046	Configural vs metric	13.68	.003	.002	.002	9	.134
Scalar	199.86	86	.930	.059	.046	Metric vs scalar	25.8	.010	.002	< .001	9	.002

Note. CFI = Comparative fit index; RMSEA = Steiger-Lind root mean square error of approximation; SRMR = Standardised root mean square residual.

### Composite reliability

Composite reliability of CD-RISC-10 scores was adequate in women (ω = .87), men (ω = .91), and the total sample (ω = .89).

### Confirmatory factor analysis of the PTG-8 and PTG-10 scales

CFA indicated that fit of the 1-factor model of the 10-item PTGI-SF scale was more or less acceptable: χ^2^/df = 180.29/35 = 5.15, RMSEA = .104 (90% CI = .089, .119), SRMR = .036, CFI = .953, TLI = .940. When adding a correlation between residuals of items 1 and 2, the fit indices improved as follows: χ2/df = 118.70/34 = 3.49, RMSEA = .08 (90% CI .065, .096), SRMR = .031, CFI = .973, TLI = .964. The convergent validity for this model was good, as AVE = .64. CFA indicated that fit of the 1-factor model of the 8-item PTGI-SF scale was more or less acceptable: χ^2^/df = 84.03/20 = 4.20, RMSEA = .091 (90% CI .071, .112), SRMR = .035, CFI = .972, TLI = .961. When adding a correlation between residuals of items 8 and 19, the fit indices were excellent as follows: χ2/df = 34.61/19 = 1.82, RMSEA = .046 (90% CI .020, .070), SRMR = .018, CFI = .993, TLI = .990. The convergent validity for this model was good, as AVE = .63. The standardised estimates of factor loadings were all adequate for both scales (see Table 4).

**Table 4:** Items of the 10-item and 8-item PTGI-SF scales in English and factor loadings derived from the confirmatory factor analysis (CFA) in the total sample.

Item	10-item PTGI-SF	8-item PTGI-SF
1. I changed my priorities about what is important in life.	.76	
19. I learned a great deal about how wonderful people are.	.68	.66
2. I have a greater appreciation for the value of my own life.	.82	.82
5. I have a better understanding of spiritual matters.	.73	.72
7. I established a new path for my life.	.84	
8. I have a greater sense of closeness with others.	.69	.66
18. I discovered that I’m stronger than I thought I was.	.86	.86
17. I have a stronger religious faith.	.82	.83
11. I am able to do better things with my life.	.88	.88
10. I know better that I can handle difficulties.	.89	.90

### Gender invariance

As reported in Table 5, all indices suggested that configural, metric, and scalar invariance of both 10-item and 8-item PGTI-SF was supported across gender. Given these results, we computed an independent-samples t-test to examine gender differences in terms of both scores. The results showed that there was no statistically significant difference between men and women in terms of 10-item PGTI-SF (M = 27.78, SD = 11.59 vs M = 28.23, SD = 11.93; t(385) = .377, p = .707, d = 2.32) and 8-item PGTI-SF (M = 22.24, SD = 9.22 vs M = 22.65, SD = 9.52; t(385) = .428, p = .669, d = 2.89).

**Table 5:** Measurement invariance across gender in the total sample.

Model	χ^2^	df	CFI	RMSEA	SRMR	Model Comparison	Δχ^2^	ΔCFI	ΔRMSEA	ΔSRMR	Δdf	p
Model 1: 10-item PTGI-SF												
Configural	157.88	68	.971	.059	.024							
Metric	178.76	77	.968	.059	.033	Configural vs metric	20.88	.003	< .001	.009	9	.013
Scalar	206.37	86	.962	.060	.034	Metric vs scalar	27.61	.006	.001	.001	9	.001
Model 2: 8-item PTGI-SF												
Configural	72.52	38	.985	.049	.025							
Metric	89.75	45	.981	.051	.034	Configural vs metric	17.23	.004	.002	.009	7	.016
Scalar	110.93	52	.975	.054	.037	Metric vs scalar	21.18	.006	.003	.003	7	.003

Note. CFI = Comparative fit index; RMSEA = Steiger-Lind root mean square error of approximation; SRMR = Standardized root mean square residual.

### Composite reliability

Composite reliability of scores was adequate for 10-item PTGI-SF in women (ω = .95), men (ω = .95), and the total sample (ω = .95). Same goes for 8-item PTGI-SF in women (ω = .93), men (ω = .94), and the total sample (ω = .93).

### Construct validity

Higher resilience was significantly and positively correlated with higher PTG. Both scores were significantly and moderately-highly associated with higher cognitive reappraisal and lower emotion suppression (Table 6).

**Table 6:** Correlations of CD-RISC-10, 10-item and 8-item PTGI-SF with the other measures on the total sample.

	1	2	3	4	5	6
1. Resilience	1					
2. 10-item PTGI-SF	.47***	1				
3. 8-item PTGI-SF	.46***	.99***	1			
4. Cognitive reappraisal	.47***	.60***	.59***	1		
5. Emotion suppression	-.41***	-.46***	-.46***	-.74***	1	
6. Age	-.07	-.01	-.01	-.03	.01	1

***p < .001

## Discussion

The main objective of the current study was to examine the psychometric properties of an Arabic translation of the CD-RISC-10, the 10-item and the 8-item PTGI-SF in Arabic-speaking community adults in Lebanon. Overall, our findings demonstrated that these three forms had unidimensional factor structure, good reliability, convergent validity and gender invariance, suggesting they can be used to investigate resilience and PTG in Arabic-speaking people, at least in the Lebanese context. Over the last years, Lebanon has known unprecedented social, political and economic crises, a resurgence of violent extremism, with a string of disasters (e.g., the Beirut blast) [95–97], making the country a unique context to investigate trauma responses. Providing the Arabic CD-RISC-10 and PTGI-SF, adapted to the Lebanese context, may allow the identification of individuals who should be targeted by interventions based on low resilience and PTG levels.

The current analyses revealed that all 10 items of the Arabic CD-RISC-10 were represented by a common factor and had an excellent internal reliability, thus confirming the robust psychometric characteristics of the measure. These findings are in line with the original study [28], as well as other validation studies that examined the psychometric quality and usefulness of the CD-RISC-10 in different sub-samples, i.e. Chinese adolescent students [35], Canadian [29], Spanish [30], Nigerian [31], and US [36] university students, Finnish [32], Spanish, Chinese [39], and Persian older adults [34]. Similarly, the Arabic 10-item and the 8-item PTGI-SF yielded single-factor structure and showed adequate internal consistency, supporting their unidimensionality and excellent psychometric properties as observed in the original validation studies [42, 43], and other adaptations of the scales in various contexts and cultural backgrounds (e.g., [43, 46–52]).

As expected, our data supported the assumption of measurement invariance across gender groups at the configural, metric, and scalar levels for all three scales. Some previous studies suggested that gender invariance of the CD-RISC-10 is not established [36]. For instance, women from a community US sample [98] and a Canadian medical students sample [99] displayed lower resilience scores. Likewise, another research found that US female public accounting had lower resilience compared to males, but also provided evidence of validity and stability of the scale factor structure across gender groups [100]. Other researchers, however, documented no differences by gender in total CD-RISC-10 scores [32]. Regarding the PTGI-SF, differences across gender have also been observed, with women reporting greater PTGI levels [46, 101, 102], whereas some evidence supported its invariance across gender (e.g. [46, 49]).

To test the divergent validity of all three scales, we explored the correlations of the CD-RISC-10, 10-item and 8-item PTGI-SF total scores with the two emotion regulation dimensions. We found that higher resilience and higher growth significantly correlated with greater cognitive reappraisal and lower emotion suppression, suggesting a high discriminant validity. This is in agreement with prior research suggesting that resilient people who manifest positive changes and growth in the face of adversity are more likely to regulate their emotions timely and effectively, and that emotion regulation strategies may foster adaptive positive responses, such as resilience and PTG [103]. Using emotion regulation strategies has also been shown to be potential mechanism used by resilient people as buffers against stressful events [104, 105]. In sum, our understanding of the dynamics of these correlations is relevant evidence to the distinctive nature of the resilience, growth and emotion regulation concepts [103].

### Study limitations

Our findings should be considered as preliminary due to certain limitations. The study has a cross-sectional design and a self-report nature. The study was conducted in one Arab country, Lebanon, limiting any generalization of our findings to Arab-speaking people from other Arab and non-Arab countries. Future longitudinal and cross-cultural research is needed to address these limitations. A selection bias is present because of the snowball sampling technique used to recruit participants and the fact that most participants had a university level of education, which limits the generalizability of the results. Participants were expected to read through inclusion criteria and agree that they were residents and citizens of Lebanon, however, there is a possibility of non-citizens/residents having potentially filled out the questionnaire. The single-factor CD-RISC-10 exhibited borderline convergent validity, which points to the need for further adaptation of the measure for other contexts. Further research needs to address other psychometric properties of the scales, namely test-retest reliability, convergent and divergent validity, as well as content and criterion-validity (predictive validity). Finally, there is a need to validate further measures that apply the social-ecological resilience theory, and account, therefore, for complex linkages of individuals, families, communities, and the cultural environment in fostering resilience (such as the student social-ecological resilience measure (Student-SERM) [106]).

## Conclusion

The present findings add to the existing literature by providing three brief and psychometrically valid measures of resilience and PTG in the Arabic language (i.e., the CD-RISC-10, the 10-item and the 8-item PTGI-SF). Beyond their good psychometric characteristics, the brevity of these three scales offers major advantages for Arab investigators and respondents in terms of reduced data collection time, burden and costs. We preliminarily suggest that these Arabic instruments are appropriate for use in Lebanese community adults to assess different positive responses after life crises, identify people with lack or low levels of resilience and growth who might need intervention, and monitor their response to therapy. Further cross-cultural validations should seek to extend their use in broader Arabic-speaking populations and settings.

## Supporting information

S1 Checklist STROBE statement—checklist of items that should be included in reports of observational studies. (DOCX)
